# Associated Mirror Therapy Enhances Motor Recovery of the Upper Extremity and Daily Function after Stroke: A Randomized Control Study

**DOI:** 10.1155/2021/7266263

**Published:** 2021-09-29

**Authors:** Jin-Yang Zhuang, Li Ding, Bei-Bei Shu, Dan Chen, Jie Jia

**Affiliations:** ^1^Department of Rehabilitation Medicine, Huashan Hospital, Fudan University, Shanghai, China; ^2^Department of Rehabilitation Medicine, Shanghai Jing'an District Central Hospital, Shanghai, China; ^3^National Clinical Research Center for Aging and Medicine, Huashan Hospital, Fudan University, China

## Abstract

Bimanual cooperation plays a vital role in functions of the upper extremity and daily activities. Based on the principle of bilateral movement, mirror therapy could provide bimanual cooperation training. However, conventional mirror therapy could not achieve the isolation of the mirror. A novel paradigm mirror therapy called associated mirror therapy (AMT) was proposed to achieve bimanual cooperation task-based mirror visual feedback isolating from the mirror. The study was aimed at exploring the feasibility and effectiveness of AMT on stroke patients. We conducted a single-blind, randomized controlled trial. Thirty-six eligible patients were equally assigned into the experimental group (EG) receiving AMT and the control group (CG) receiving bimanual training without mirroring for five days/week, lasting four weeks. The Fugl-Meyer Assessment Upper Limb subscale (FMA-UL) for upper extremity motor impairment was used as the primary outcome. The secondary outcomes were the Box and Block Test (BBT) and Functional Independence Measure (FIM) for motor and daily function. All patients participated in trials throughout without adverse events or side effects. The scores of FMA-UL and FIM improved significantly in both groups following the intervention. Compared to CG, the scores of FMA-UL and FIM were improved more significantly in EG after the intervention. The BBT scores were improved significantly for EG following the intervention, but no differences were found in the BBT scores of CG after the intervention. However, no differences in BBT scores were observed between the two groups. In summary, our study suggested that AMT was a feasible and practical approach to enhance the motor recovery of paretic arms and daily function in stroke patients. Furthermore, AMT may improve manual dexterity for poststroke rehabilitation.

## 1. Introduction

Stroke is a leading cause of mortality and long-term disability worldwide [1], which results in a global economic burden for health care [2, 3]. Currently, many advanced technologies have been worked out and used for stroke survivors. Nevertheless, we still face many challenges for poststroke rehabilitation, for instance, the paretic upper extremity. After stroke, about 80% of patients remain having upper extremity motor impairment [4]. Besides, researchers have found that the ipsilesional upper limb also suffered motor dysfunction in 3 months after the onset of stroke [5], which hinders physical function and independent daily activities.

Compared to the healthy population, stroke patients tend to avoid bilateral motor patterns in daily activities [6]. Lots of daily activities are inseparable from bimanual cooperation, such as twisting the towel, driving the car, and getting dressed. For this reason, bilateral task relearning is essential for stroke patients. However, most therapeutic methods for stroke are concentrated on improving the contralesional arm function, ignoring participation of the less affected side [7–9]. It is remarkable that protocols of bilateral treatment (BT) which involve bilateral training with rhythmic auditory cues, bilateral priming, and device-driven bilateral training have been used as clinical treatments for stroke rehabilitation [10–12]. Based on bilateral, repetitive, and symmetrical motor principles, most bilateral treatments (BTs) are executed through two independent and paralleled actions, which ignore cooperation between the hands; for instance, Sainburg et al. proposed the symmetrical cooperative tasks regarded as a bilateral synergy framework for poststroke rehabilitation [6].

In addition to conventional physical intervention methods, mirror therapy (MT) which relies on visual illusion is regarded as a bilateral treatment [13, 14]. Under the MT environment, a plane mirror is placed in the median sagittal plane between upper limbs to induce the visual illusion, and patients are asked to move both arms as far as possible. Contrary to viewing directly on both arms, MT can provide normal visual stimulation of bilateral movement, which has been proven to promote better the activation and functional connectivity in the somatosensory system of the brain [15]. In addition, better than most protocols of BT, MT may have a priming effect on motor recovery through mirror illusion [14, 16]. Hence, compared to conventional BTs, MT may be a superior approach for bilateral task relearning for stroke patients. Following the types of action, the protocols of MT contain manipulation of objects, manipulation without objects, and both in combination [17]. However, relying entirely on a plane mirror or “mirror box,” the conventional protocol of MT cannot achieve it for isolating two hands from the mirror and only provides unilateral visual feedback. Due to the limitations, manipulation of objects under MT cannot attain bimanual cooperation and may affect the priming activation of mirror visual feedback (MVF) [18–20]. Besides, the poor posture in the conventional MT procedure can easily cause pressure on the spine and impede effective bimanual cooperation relearning [21].

To overcome the limitations of the traditional MT, researchers have proposed novel mirror setups. Camera technique-based MVF, which offered bilateral visual feedback, was one of those, and previous researches have been verified that camera technique-based MVF can promote the functional recovery of stroke [22–24].

We previously put forward a novel camera technique-based MVF with an operable mirror environment [21]. Patients can achieve synchronous movement of both upper extremities isolated from the mirror in such an environment. Previous studies have proven its clinical feasibility and effectiveness for stroke rehabilitation [21, 25]. Based on the setup, we developed a novel MT paradigm, in which both upper extremities were associated with one object, and patients were asked to complete the same tasks to realize the association of both sides. In the paradigm of MT, we named it associated mirror therapy (AMT). We conducted a randomized controlled trial to certificate AMT's feasibility and clinical efficacy, and we hypothesized that AMT could promote the recovery of the paretic upper extremity and daily function for stroke patients.

## 2. Methods

### 2.1. Study Design

This study was an assessor-blinded, pretest-posttest, randomized controlled trial. A separate investigator was responsible for the clinical assessments but blinded to the allocation. Meanwhile, two occupation therapists who were responsible for the therapeutic regimens were trained by one researcher. All patients received assessments before the intervention, after 2-week and 4-week intervention. The study was approved by the ethics committee institutions of Huashan Hospital (KY2017-230) and registered at the Chinese Clinical Trial Registry (ChiCTR1800018351).

### 2.2. Participants

Patients were recruited from the Department of Rehabilitation Medicine, Huashan Hospital Affiliated Jing'an Branch. Patients who had a first-ever ischemic or hemorrhagic stroke, occurring three months to one year, aged between 25 and 75 years without severe cognitive impairment (Mini‐Mental State Examination (MMSE) score > 24), were included. All patients were within the Brunnstrom stage of hand over III and with modified Ashworth scale ≤ 2. Patients who met any of the following conditions were excluded: (i) the condition deteriorated during the intervention; that is, the stroke relapsed or a new infarction occurred; (ii) psychiatric disorder or other serious illness that interfered with the patients' ability to obey the therapists; (iii) and having experienced other central intervention methods, for instance, transcranial direct current stimulation (tDCS). The enrolled patients were given written informed consent before the study.

**Figure 1:**
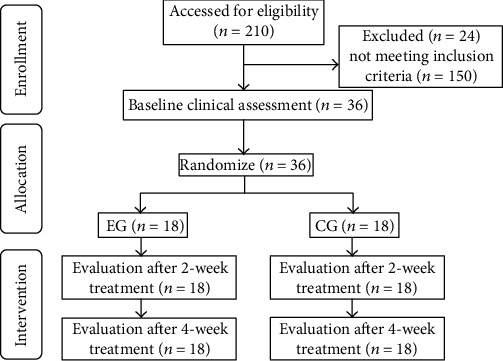
The flowchart of recruiting patients.

We speculated that the primary outcome (FMA-UL) had a group × time interaction. Based on the previous camera technique-based MVF studies [21, 25, 26], an effect size of 0.27 to 0.45 was expected to detect the differences in the improvements between groups. Given the reliability and safety margin, an effect size of 0.27 was anticipated for repeated analysis of variance (ANOVA). Then, we estimated a total sample size of 30 which was needed for providing 80% power to detect the differences between groups on FMA-UL with a type I error of 0.05 and a dropout rate of 20%. In addition, we reviewed all published clinical trials on MT, and the sample size of most studies ranged from 10 to 20 patients in each group. Therefore, we planned to recruit 18 patients in each group. The process of recruiting patients is shown in Figure 1.

### 2.3. Randomization and Allocation

Eligible patients were randomly assigned to the control group (CG) and experimental group (EG). An independent researcher executed the randomization procedure, generated through a random data generator on the computer. A sealed envelope was used to confirm the group of each patient who was satisfied with recruitment criteria. When receiving an envelope, therapists were informed to perform the patient assignment.

### 2.4. Intervention

All enrolled patients received the conventional stroke rehabilitation program for four weeks, five days/week, and around four hours/day. The conventional stroke program consisted of physiotherapy, occupation therapy, speech therapy, and respiratory management.

#### 2.4.1. Experimental Group

*(1) Setup*. The setup (1200 mm × 940 mm × 702 mm) was mounted with a 23.8-inch light-emitting diode screen of 30° tilt, fixed on the mirror setup to present the mirror image, and blocked the direct view of both hands [21]. Two cameras were mounted on the top of the mirror setup to capture the movements of the hands. In the mirrored environment, patients were allowed to put both hands on the bottom of the “mirror box”, of which one side opening was beneath the screen. The therapist could assist the patients on the other side. Patients could sit in a suitable and comfortable position by adjusting the setup height during the treatment.

**Figure 2:**
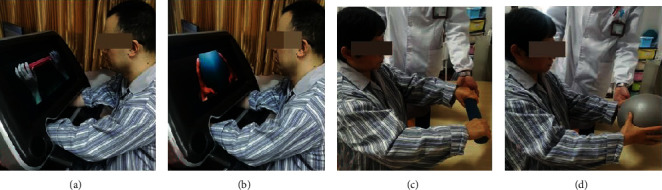
AMT and conventional bimanual training for stroke rehabilitation: (a, b) AMT: grabbing and rolling a cylinder/holding a ball; (c, d) conventional bimanual training: grabbing and rolling a cylinder/holding a ball.

*(2) Associated Mirror Therapy (AMT)*. Based on the above setup, we created a novel clinical paradigm of MT, in which patients not only could see the regular bimanual cooperation but also could attain the bilateral cooperative tasks with the assistance of therapists. In the paradigm, both upper extremities were associated with the identical object and completed synchronously the same task, e.g., holding a ball, grabbing and rolling a cylinder, stacking of towels, lifting a stick, and pushing a sanding board (see Figures 2(a) and 2(b)). Patients were required to focus on the screen and imagined doing cooperative tasks with both arms. Meanwhile, patients were asked to perform the same training synchronously by the affected side as much as possible. During the trial, therapists offered essential directions to make patients concentrate on the screen and immerse themselves in mirror illusion. Another role of therapists was to supervise and ensure the completion of actual bilateral cooperative tasks of patients. Conforming to the motion of the less affected arm of the patient, the therapist could provide active, assisted, or passive movement for the affected side alternatively. We named the novel paradigm “associated mirror therapy” (AMT) for achieving a practical bimanual interaction under camera technique-based MVF.

In addition to the conventional rehabilitation, patients in EG received half an hour of AMT firstly. Based on the patient's condition, therapists selected 2 to 3 kinds of bilateral cooperative tasks. Subsequently, another half-hour upper limb training was applied, including stretching, relaxing, and functional activities.

#### 2.4.2. Control Group

Patients in CG received the same dose of training as EG. However, the only difference was that CG received bimanual cooperation training without camera technique-based MVF, where patients had a direct view of both arms. To assure the performance of bilateral cooperative tasks, therapists also provided necessary assistance to help patients (see Figures 2(c) and 2(d)).

### 2.5. Assessments

The basic information, including age, sex, lesion side of the brain, stroke type, and duration after stroke onset, was recorded. The clinical outcomes were concerned with motor impairment, motor function, and daily function. The outcome measures were evaluated repeatedly before the intervention, after 2-week and 4-week intervention to verify clinical efficacy. The specific evaluation details of outcome measures were as follows.

The primary outcome was the change of motor impairment measured through the Fugl-Meyer Assessment Upper Limb subscale (FMA-UL). The FMA-UL subscale with good psychometric properties indicated high reliability and validity for motor impairment [27]. The FMA-UL subscale included 33-item upper limb activities. Each item was rated on a 0 to 2 ordinal scale. The maximum score of the FMA-UL subscale was 66.

Secondary outcomes were the performances of motor and daily function. The Box and Block Test (BBT) with satisfactory reliability and validity was used to assess motor function for manual dexterity in stroke patients [28]. The BBT contains 150 colored wooden cube blocks (1 inch, 2.5 cm × 2.5 cm × 2.5 cm). The participants were told to move one-by-one blocks as many as possible from a rectangular box container to the other of equal size within 60 seconds. Both hands' scores of the BBT were calculated, respectively, by the number of blocks transferred. The Functional Independent Measurement (FIM) was widely applied to evaluate participation after stroke [29]. FIM involved six aspects of daily function: self-care, sphincter control, transfer, locomotion, communication, and social cognition ability. It was made of 18 items, and each item was graded on a 1 to 7 ordinal scale. The total score ranged from 7 to 126.

### 2.6. Statistical Analyses

The data were analyzed by using SPSS version 20.0 for Windows (SPSS Inc., Chicago, IL, USA). We used Shapiro-Wilk's test to check the underlying model assumptions for normality of distribution entirely. None of evaluation indicators satisfied the normal distribution. Baseline characteristics of the patients between groups were compared by using chi-square tests or Fisher's exact test (including gender, side of paralysis, stroke types, and Brunnstrom stage) when appropriate. The Mann–Whitney *U* test was used to examine the baseline data of continuous variables between groups (including age and course of the disease). The generalized estimating equation (GEE) model based on a binary outcome with first-order autoregressive correlation structure (AR (1)) was used to explore multitime repeated measurement analysis [30, 31], including three outcome indicators (FMA-UL, FIM, and BBT). The main effects of group, time, and group-by-time interaction were analyzed in the GEE model. A value of *P* < 0.05 was considered significant.

## 3. Results

### 3.1. Baseline Characteristics

From October 2018 to August 2019, 36 stroke patients were recruited, with 18 patients in each group. All patients completed the trial without side effects, and adverse events occurred during the trial.

The clinical characteristics of the two groups of patients were demonstrated in Table 1. The median age (QR) was 54.0 (24.00) and 58.0 (22.75) years for EG and CG, respectively (*P* = 0.350), with no difference in sex between groups. No significant difference was found in the course of stroke (*P* = 0.198). There were no differences in the type and location of stroke between EG and CG (*P* = 0.725, *P* = 0.738). No differences were found between the two groups in the Brunnstrom stages of the proximal and distal areas of the affected upper extremity (*P* = 0.464, *P* = 0.876).

### 3.2. Treatment Effects on Clinical Outcomes

The improvements of paretic arm impairment and daily function were observed in both groups. Manual dexterity had a significant change in EG after the intervention, whereas the improvement did not occur in CG. Significant group-by-time interaction effects were found in FAM-UL scores (Wald_*χ*^2^_ = 174.434, *P* < 0.001), BBT scores (Wald_*χ*^2^_ = 18.594, *P* = 0.002), and FIM scores (Wald_*χ*^2^_ = 100.165, *P* < 0.001) after the intervention; therefore, the single group or time effect estimate was not applicable during the study (see Table 2). The treatment effects of clinical outcomes are shown in Tables 3–5. The detailed comparisons between the two groups were reported below.

After the 4-week trial, FMA-UL scores in both groups were significantly higher than before (*P* < 0.001 and *P* < 0.001, respectively). Both EG and CG had a continuous improvement in FMA-UL scores over time, including the first two weeks (*P* < 0.001 and *P* < 0.001, respectively) and the last two weeks of intervention (*P* < 0.001 and *P* < 0.001, respectively). Post hoc analyses indicated no difference in FAM-UL between EG and CG before the trial (*P* = 0.290). Moreover, the scores of FMA-UL in the EG were significantly higher than those in the CG after 2 and 4 weeks (*P* = 0.018 and *P* = 0.001, respectively).

Significant improvements of BBT scores in the first and the last two weeks were observed in EG (*P* = 0.002 and *P* < 0.001). After the intervention, BBT scores in the EG were significantly improved (*P* < 0.001). However, in the CG, only in the last two weeks, the BBT scores were significantly improved (*P* = 0.043). After the 4-week intervention, no difference in BBT scores was observed in the CG than before (*P* = 0.107). By comparing EG with CG, no differences in BBT scores were observed before the trial, after 2-week and 4-week intervention (*P* = 0.780, *P* = 0.569, and *P* = 0.377, respectively). Although the difference in manual dexterity measured by BBT scores was not significant between both groups, a clinical improvement is in favor of AMT.

After the 4-week trial, FIM scores in both groups were significantly higher than before (*P* < 0.001 and *P* < 0.001, respectively). A significant improvement of FIM scores in the first and the last two weeks was observed in the EG (*P* < 0.001 and *P* < 0.001, respectively). FIM scores in the CG were also improved in the first and the last two weeks (*P* < 0.001 and *P* = 0.006, respectively). When compared between groups, no difference in FIM scores was observed before the trial (*P* = 0.287). However, improvement of FIM scores in the EG was better than the CG after 2 and 4 weeks (*P* = 0.041 and *P* = 0.003, respectively).

## 4. Discussion

In the present study, we proposed a novel paradigm of MT, called AMT, which achieved bimanual cooperation under camera technique-based MVF. Besides, we testified to the feasibility and effectiveness of AMT. All patients participated in trials throughout without adverse events or side effects, proving that the AMT was safe and feasible. The study demonstrated that using AMT as an auxiliary therapy to usual care could decrease the motor impairment of the paretic upper extremity and enhance daily function for stroke patients. In addition, AMT may increase manual dexterity after stroke.

The coordinated control was also regarded as an essential function of the standard upper extremities, especially for instrumental activities of daily living (IADL) [32–34]. After stroke, patients lacked the participation of the paretic upper extremity in daily tasks [35]. Previous studies combined MT with daily functional activities, demonstrating that the MT paradigms could enhance the motor recovery of the paretic upper extremity in stroke patients [36, 37]. Although many protocols of MT were proposed, few of those achieved practical bimanual tasks to associate both upper extremities. Rodrigues et al. developed the bilateral task-based MT, which related both arms to one object under the mirror environment [38]. As far as we know, this was the first study to propose bilateral tasks based on MT. Despite proving the feasibility of combining bilateral symmetrical tasks with MT, patients were asked to concentrate on the reflection side of the mirror and could not ensure the participation of the paretic side. New setups for MT conquered the limitations of the conventional mirror, for instance, the camera technique-based MT proposed by Lee et al., which realized MVF effect delay and bilateral movements [22, 24]. We previously put forward novel camera technique-based MVF [21]. To achieve bimanual coordination control under the mirror, we designed bimanual cooperation tasks in which both arms were associated with one object and completed the same tasks synergistically.

Compared to usual care, camera technique-based MVF was proven to enhance the motor impairment of the upper extremity after stroke [22, 25]. In line with the results of previous studies, the improvements in motor impairment measured by FAM-UL were observed in both groups after the intervention, and patients in the EG were improved more significantly than the CG. Furthermore, compared with bilateral arm training, researchers also found a more significant improvement for the distal arm which was in favor of MT [39]. Our results were similar to the above study. Previous studies revealed that MVF might have the potential to promote motor learning by activating neural areas related to spatial attention, which was beneficial to enhance the perception of the paretic arm [40, 41]. Therefore, compared to conventional BT, the result might be interpreted that MVF activated the related sensorimotor brain area through visual illusion. Previously, Rodrigues et al. put forward adding an object to the plane mirror to realize bilateral symmetrical training under MT [38]. Researchers discovered that no differences were found between bilateral symmetrical tasks with or without MT. Our results were different from it. Noticeably, the MT paradigm in our study was different from the one Rodrigues et al. proposed. The main difference was that we used camera technique-based MVF rather than a plane mirror. In addition, in the present study, patients could accomplish practical bimanual tasks under the therapist's assistance, but the paretic upper extremity's quality could not be guaranteed in the above conventional MT paradigm.

When it came to motor function, one previous study pointed out that MT could promote the manual dexterity of stroke patients evaluated by BBT [42]. Our finding was similar to the study. In the comparison before and after the intervention, there was no difference of manual dexterity in CG. However, a significant effect of gross hand dexterity measured by BBT was found in AMT after the intervention. Although the difference in motor function improvements between both groups was not statistically significant, the performance was better in AMT. Besides, interestingly, a sustained motor function improvement of the upper extremity changed with time in EG, but not in CT. This phenomenon might be caused where AMT had a sustained regular visual input of movement, which may better promote central brain remodelling [15]. Our previous studies revealed that camera technique-based MVF could activate motor preparation and brain network segregation by inducing mirror illusion, which might promote motor execution for stroke patients [21, 25].

In this study, we used bilateral cooperation tasks with or without camera technique-based MVF for stroke patients. All patients had gained significant improvement in daily function after the trial, in line with previous studies of BT or MT [43, 44]. In addition, previous studies showed that MT was more effective than conventional methods in improving the daily function of stroke patients [36, 45]. Our result was similar to the above conclusion. A more significant improvement in daily function was observed in AMT when comparing the differences between both groups. It might be related to the more significant improvement of motor impairment of paretic arms in EG after the intervention. The daily function is related closely to the upper extremity function, for instance, self-care. However, in the present study, we did not compare the improvements between both groups in different aspects of daily function based on FIM. Then, the improvements of specific daily functions in AMT were unknown.

The study has some limitations. Firstly, the novel paradigm relied on camera technique-based MVF which is labour intensive. Secondly, though we have estimated the sample size, the results should be considered cautiously because of the small sample size. Thirdly, we only conduct the 4-week intervention without follow-up, and the long-term and sustained intervention effects for stroke patients are unknown. Hence, we will search for a more convenient and economical method of AMT by equipment upgrade. In addition, a larger RCT should be conducted to certify further the long-term and sustained effects of AMT on stroke patients.

## 5. Conclusions

In summary, this is the first study to propose a novel and advantageous MT paradigm achieving bimanual cooperation under camera technique-based MVF. The present study demonstrates that AMT is a feasible and effective method to improve motor impairment of the paretic arm, enhance daily function, and may increase the ability of manual dexterity after stroke.

## Figures and Tables

**Table 1:** Characteristics of study participants (*n* = 36).

Variable	EG (*n* = 18)	CG (*n* = 18)	*P* value
Age (years), *M* (QR)	54.0 (24.00)	58.0 (22.75)	0.350
Sex, *N*			
Male/female	12/6	12/6	1.000
Lesion side, *N*			
Left/right	9/9	10/8	0.738
Stroke type, *N*			
Hemorrhagic/schemic	13/5	11/7	0.725
Months after stroke onset, *M* (QR)	4.0 (5.25)	5.0 (7.25)	0.198
Brunnstrom (3/4/5/6), *N*			
Distal	12/2/2/2	14/1/1/2	0.876
Proximal	12/2/2/2	16/1/0/1	0.464

EG: experimental group; CG: conventional group.

**Table 2:** Description for group effect, time effect, and group × time effect on motor impairment, motor function, and daily function.

Outcomes	Group		Time		Group × time	
	Wald_*χ*^2^_	*P*	Wald_*χ*^2^_	*P*	Wald_*χ*^2^_	*P*
FAM-UL	4.858	0.028	141.058	<0.001	174.434	<0.001
FIM	3.893	0.048	58.687	<0.001	100.165	<0.001
BBT	0.192	0.662	17.310	<0.001	18.594	0.002

FAM-UL: Fugl-Meyer Assessment Upper Limb subscale; BBT: Box and Block Test; FIM: Functional Independence Measure.

**Table 3:** Description and comparison between groups for statistical outcomes on motor impairment, motor function, and daily function.

Outcomes	Pretest			After 2 weeks			After 4 weeks		
	EG	CG	*P*	EG	CG	*P*	EG	CG	*P*
FMA-UL	32.5 (25.50)	28.0 (11.00)	0.290	41.5 (13.25)	30.0 (11.75)	0.018	45.0 (22.50)	30.5 (13.50)	0.001
FIM	108.0 (8.00)	104.5 (15.00)	0.287	111.(8.50)	106.0 (15.50)	0.041	113.5 (8.50)	107.0 (14.50)	0.003
BBT	0.5 (12.00)	0.0 (3.00)	0.780	2.0 (21.50)	0.0 (3.25)	0.569	3.0 (24.00)	0.0 (6.25)	0.377

EG: experimental group; CG: conventional group; FAM-UL: Fugl-Meyer Assessment Upper Limb subscale; BBT: Box and Block Test; FIM: Functional Independence Measure.

**Table 4:** Description for motor impairment, motor function, and daily function in EG.

Outcomes	Pretest	After 2 weeks	After 4 weeks	*P*
FMA-UL	32.5 (25.50)	41.5 (23.25)^a^	45.0 (22.50)^b^	<0.001
FIM	108.0 (8.00)	111.0 (8.50)	113.5 (8.50)	<0.001
BBT	0.5 (12.00)	2.0 (21.50)	3.0 (24.00)	<0.001

^a^Comparison between pretest and after 2-week intervention. *P*_FMA‐UL_ < 0.001, *P*_FIM_ < 0.001, and *P*_BBT_ = 0.002. ^b^Comparison between after 2-week intervention and after 4-week intervention. *P*_FMA‐UL_ < 0.001, *P*_FIM_ < 0.001, and *P*_BBT_ < 0.001. EG: experimental group; FAM-UL: Fugl-Meyer Assessment Upper Limb subscale; BBT: Box and Block Test; FIM: Functional Independence Measure.

**Table 5:** Description for motor impairment, motor function, and daily function in CG.

Outcomes	Pretest	After 2 weeks	After 4 weeks	*P*
FMA-UL	28.0 (11.00)	30.0 (11.75)^c^	30.5 (13.50)^d^	<0.001
FIM	104.5 (15.00)	106.0 (15.50)	107.0 (14.50)	<0.001
BBT	0.0 (3.00)	0.0 (3.25)	0.0 (6.25)	0.107

^c^Comparison between pretest and after 2-week intervention. *P*_FMA‐UL_ < 0.001, *P*_FIM_ < 0.001, and *P*_BBT_ = 1. ^d^Comparison between after 2-week intervention and after 4-week intervention. *P*_FMA‐UL_ < 0.001, *P*_FIM_ = 0.006, and *P*_BBT_ = 0.043. CG: conventional group; FAM-UL: Fugl-Meyer Assessment Upper Limb subscale; BBT: Box and Block Test; FIM: Functional Independence Measure.

## Data Availability

We have utilized an unauthorized translation of the Chinese MMSE in the research, and we have received the permission of Psychological Assessment Resources (PAR). The study data are available from the corresponding author.
